# Supplementary material for: Feasibility of Muscle Endurance Testing in Critically Ill Trauma Patients: A Pilot Study
Source: Healthcare (Basel). 2022 Dec 24;11(1):53. doi: 10.3390/healthcare11010053 (PMC9818946; doi:10.3390/healthcare11010053)
Supplement: Supplementary file 1 [file healthcare-11-00053-s001.zip › healthcare-2008080-supplementary.pdf]

Safety Criteria for Early Mobilization in ICU

Number		Name		Sex		Date	

PASS	☐	FAIL	☐

System	Screening Item	
Cardiovascular	☐ SBP < 90mmHg or SBP > 180mmHg<br>☐ MBP < 60mmHg or MBP > 110mmHg<br>☐ HR < 50bpm or HR > 130bpm<br>☐ New or symptomatic arrhythmia, MI<br>☐ PTE (discuss with MD to determine suitability)<br>☐ DVT (may mobilize as tolerated immediately after LMWH is given)<br>☐ Platelet count < 20,000/uL	< Vasoactive agent ><br>: new vasoactive agent, use of three or more agents or frequent increases<br>☐ Dopamine ☐ Norepinephrine<br>☐ Vasopressin ☐ Epinephrine<br>☐ Other :
Respiratory	☐ RR ≤ 5 or RR ≥ 40breaths/min ☐ SpO_2 ≤ 88% ☐ FiO_2 ≥ 0.6 ☐ PEEP > 10mmHg<br>☐ Airway is not adequately secured	
Neurologic	☐ Severe agitation, stress state ☐ Attention required for increased intraocular pressure<br>☐ ICP ≥ 20mmHg ☐ Unstable spinal injury or lesion ☐ CSF leakage or lumbar drain	
Other	☐ BT ≥ 38˚C ☐ Active or uncontrolled bleeding ☐ Unstable fracture ☐ Open abdomen	

*the cited values are not absolute criteria for withholding mobilization or chest physiotherapy. It may be modified according to the medical condition of the critically ill patient.

Stop Criteria

☐ Abnormalities in the above-mentioned vital signs occur. (If it is not stabilized even after stopping mobilization.)

☐ Changes in consciousness	☐ New/symptomatic arrhythmia
☐ Ventilator asynchrony	☐ Fall
☐ Bleeding	☐ Medical device removal or malfunction
☐ Distress reported by patient or observed by clinician	

Safety Fail Event :*(comment field)*
